# RNA-Binding Protein La Mediates TGFβ-Induced Epithelial to Mesenchymal Transition and Cancer Stem Cell Properties

**DOI:** 10.3390/cancers13020343

**Published:** 2021-01-19

**Authors:** Tilman Heise, Gunhild Sommer

**Affiliations:** Department for Pediatric Hematology, Oncology and Stem Cell Transplantation, University Hospital Regensburg, Franz-Josef-Strauss Allee 11, 93053 Regensburg, Germany; tilman.heise@ukr.de

**Keywords:** La protein, La/SSB, LaRP3, RNA-binding protein, RBP, plasticity, TGFβ, EMT, cancer stem cells, CSC, AKT, phosphorylation

## Abstract

**Simple Summary:**

Reversible epithelial to mesenchymal transition (EMT) plays a key role in establishing a malignant phenotype by assuring cancer cell plasticity critical for cancer progression by allowing a small fraction of cancer cells to detach from primary lesions and outgrow at metastatic sites. Cancer cell plasticity is associated with cancer stem cell properties contributing to chemoresistance, metastasis, and poor clinical outcomes. Dysregulated RNA-binding proteins are key players in controlling the RNA metabolism, including mRNA processing, export, and translation, and have been implicated in cancer cell plasticity. In this study, we demonstrated that aberrantly expressed RNA-binding protein La is critical for transforming growth factor β-induced EMT and for gaining cancer stem cell properties. Understanding the function of aberrant RNA-binding protein expression in cancer cell plasticity reveals prospects for identifying novel therapeutic targets.

**Abstract:**

Background: the aberrant overexpression of predominantly nuclear localizing RNA-binding protein (RBP) La contributes to proliferation, mobility, and chemoresistance of cancer cells and tumor growth in mice. Methods: studies included cancer tissue microarrays (TMAs) analyses, cancer tissue data mining, transforming growth factor β (TGFβ)-induced cancer cell plasticity studies, three dimensional sphere growth, epithelial to mesenchymal transition (EMT) assays, analysis of cancer stem cell (CSC) marker expression, and post-translational modification of cancer-associated La protein. Results: we demonstrated that significant overexpression of RBP La in lung and head and neck cancer tissue correlates with poor overall survival. Furthermore, small interfering RNA-mediated depletion of La reduced proliferation and migration of cancer cells, blocked TGFβ-induced EMT, and diminished both EMT and CSC marker expression. Rescue experiments with La wildtype but not RNA chaperone domain activity-defective La mutant increased the expression of those cancer progression markers, suggesting a critical role of La’s RNA chaperone activity in this process. La depletion in cancer cells also significantly decreased sphere growth in the presence of TGFβ. Interestingly, TGFβ treatment induced phosphorylation of La at threonine 389 (pLa^T389^) only in adherents but not in 3D growing cultures. Conclusion: our study suggests that the TGFβ/AKT/pLa^T389^ signaling pathway regulates cancer cell plasticity.

## 1. Introduction

Ribonucleic acid (RNA)-binding proteins (RBP) are critical players of the RNA metabolism and regulate transcription, processing, editing, localization, stability, and translation of RNAs. To fulfill their manifold functions RBPs localize to the nucleus, the cytoplasm, or shuttle between both compartments and often accumulate in nuclear structures such as the nucleolus or splicing speckles. Dysregulation of RBPs is frequently associated with cancer progression. The function of RBPs is widely regulated by post-translational modifications (PTMs), including phosphorylation, acetylation, methylation, ubiquitinylation, and sumoylation, and are often allow a fast response to cellular signaling events [1,2]. By binding target RNAs, the RBPs form complexes, called ribonucleoproteins (RNPs). In cancer cells, aberrant expression and dysregulated PTMs of RBPs can change the binding activity and processing of target RNAs supporting a malignant phenotype [3].

The RBP La (La-related protein 3 (LARP3), Sjogren syndrome antigen B (SSB)) belongs to the La-related protein (LARP) family, characterized by an RNA-binding domain called La motif [4,5]. Potential functions of the different LARP family members in cancer pathobiology have been recently reviewed [6]. The La protein predominantly localizes to the nucleoplasm, but also shuttles between the nucleus and cytoplasm [7]. The RBP La is overexpressed in various cancer entities including chronic myeloid leukemia, head and neck, and cervical cancer [2]. Aberrant La protein expression contributes to increased proliferation, migration, invasion, and chemoresistance of cancer cells [8,9] and supports tumor growth in mice [10]. Investigating the cellular functions revealed that La assists processing of noncoding RNA precursors (pre-transfer (pre-t)RNA, pre-micro (pre-mi)RNA) [11], but also stimulates translation of target messenger RNAs (mRNAs) encoding oncogenic factors (reviewed in: [2]). In cancer cells, La-dependent translation of target mRNAs causes increased expression of cell cycle regulator cyclin D1 [9], p53-negative regulator mouse double minute 2 (Mdm2) [12], extracellular matrix protein laminin B1 [13], and antiapoptotic factors B-cell lymphoma 2 (Bcl2) and X-linked inhibitor of apoptosis protein (XIAP) [14,15]. All of those target mRNAs encode oncogenic factors known to contribute to cancer progression and—when aberrantly expressed—often correlate with poor clinical outcomes. Recently, we identified an RNA chaperone domain (RCD) in the carboxyterminal part of RBP La that is able to unwind RNA stem-loop structures located within the 5′-untranslated region (5′UTR) of cyclin D1 and Bcl2 mRNA, hindering efficient translation in vitro and in cell-based assays [14,16], supporting a model that suggests that the RNA chaperone activity of La contributes to overexpression of oncogenic factors in cancer cells. 

Cancer cell plasticity is the capability of cancer cells to reversibly switch between epithelial and mesenchymal states in response to extracellular signals. Epithelial to mesenchymal transition (EMT) plays a key role during establishing a malignant cancer phenotype [17]. EMT is associated with higher cell motility, increased resistance to apoptosis, and cancer stem cell (CSC) properties, resulting in chemoresistance, metastasis, and poor clinical outcomes. CSCs have been identified in various cancer entities, including lung and head and neck cancer, and represent a small cell population with the ability of self-renewal, a vast differentiation potential, and high chemoresistance driving tumor recurrence and metastasis [18]. When growing in nonadherent serum-free conditions, CSCs form multicellular three-dimensional (3D) spheres [19]. Transforming growth factor β (TGFβ) is a well-known extracellular signal-inducing EMT via the activation of the canonical SMAD pathway and the noncanonical pathways, including protein kinase B (PKB)/AKT, extracellular signal-regulated kinase (ERK), and mitogen-activated protein kinase (MAPK) [20]. A distinguished intracellular master regulator of EMT is zinc finger E-box binding homeobox 1 (ZEB1), a transcription factor establishing a pro-metastatic state in cancer cells [21,22]. 

Herein, we show that the cancer-associated RNA chaperone La is required for TGFβ-induced EMT, elevated expression of transcriptional EMT master regulator ZEB1, and supports cancer stem cell properties as indicated by higher CSC marker expression and increased sphere formation and growth. Interestingly, during EMT, TGFβ stimulates—in an AKT-dependent manner—phosphorylation of La at threonine 389 under adherent but not sphere growth conditions. Overall, this study presents the first data highlighting an important role of RBP La in cancer cell plasticity.

## 2. Results

### 2.1. Overexpression of RNA-Binding Protein La Correlates with Reduced Overall Survival in Patients and Contributes to Increased Proliferation and Migration of Cancer Cells

**Figure 1:**
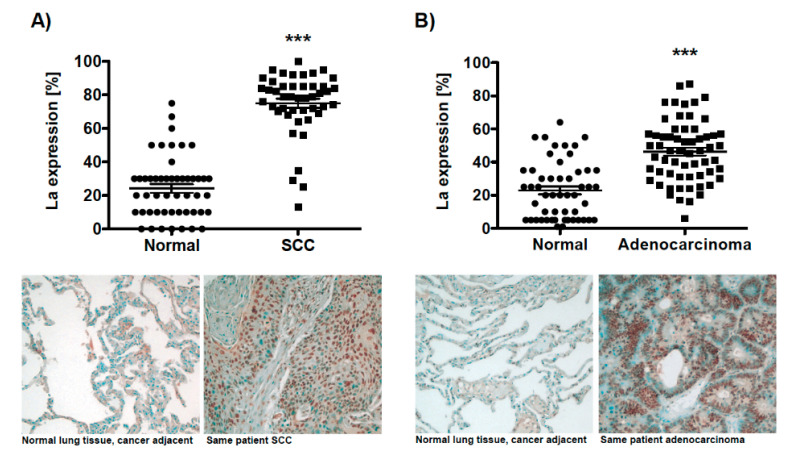
The ribonucleic acid (RNA)-binding proteins (RBP) La is overexpressed in lung cancer tissue. Immunohistochemistry of two distinct tissue microarrays (TMAs) demonstrating significant overexpression of the La protein in lung cancer tissue compared to normal cancer adjacent tissue. (**A**) Squamous cell carcinoma (normal: *n* = 50/SCC: *n* = 51; TMA BC04118), (**B**) adenocarcinoma (normal: *n* = 48/SCC: *n* = 49; TMA LC1002). Representative images of adjacent normal tissue and cancer tissue are presented, scale 100-fold magnification. The predominantly nuclear staining of the RBP La is depicted as percentages based on the largest population of benign cells present in normal tissue, respectively, tumor cells present in cancer tissue (*p* value: < 0.001 (three asterisks)).

For lung cancer, the analysis of microarray-based datasets available through the data-mining platform ONCOMINE revealed a twofold increase of La mRNA level in lung cancer tissue, which correlated with poor prognosis in non-small-cell carcinoma (NSCLC) patients [23]. To establish a more comprehensive picture of aberrant La protein expression in lung cancer, we analyzed by immunohistochemistry the La protein expression in two lung cancer tissue microarrays consisting of (A) 51 squamous cell carcinoma (SCC) along with 50 normal cancer adjacent tissues samples (TMA: BC04118) and (B) 49 adenocarcinoma along with 48 normal cancer adjacent tissues (TMA: LC1002). Our data underline a significant overexpression of the RNA-binding protein La in both SCC and adenocarcinoma lung tissues when compared to normal adjacent lung tissues (Figure 1A,B). Analyzing RBP La expression in regard to different cancer stages revealed a consistently high expression in SCC (Figure S1A) and decreased expression in higher compared to lower cancer stages in adenocarcinoma (Figure S1B). In regard to sex differences, neither in SCC nor in adenocarcinoma was a significant change of La expression observed (Figure S2A,C (male) and Figure S2B,D (female)).

**Figure 2:**
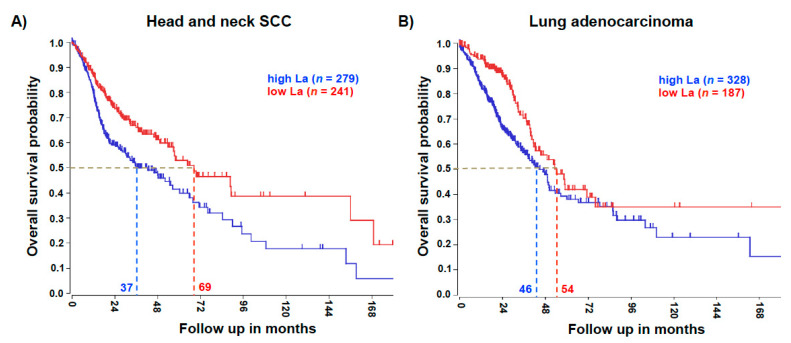
High La expression is associated with poor overall survival. (**A**) The analysis revealed that at an overall survival probability of 0.5, head and neck squamous cell carcinoma (SCC) patient survival was reduced by 32 months when La expression was high. Head and neck SCC gene expression dataset TCGA-520 was analyzed for the single La gene. A gene expression cut off of 1713 was applied; the Chi-square test (Chi = 12.02) and *p* value (*p* = 5.3 × 10^−4^) was calculated. (**B**) The analysis revealed that at an overall survival probability of 0.5, lung adenocarcinoma patient survival was reduced by 8 months when La expression was high. Lung adenocarcinoma gene expression dataset TCGA-515 was analyzed for the single La gene. A gene expression cut off of 1791 was applied; the Chi-square test (Chi = 8.63) and *p* value (*p* = 3.3 × 10^−3^) was calculated.

Significant overexpression of the La protein in head and neck SCC has been published earlier [8]. To test whether an elevated La mRNA level correlates with the poor survival of patients, we mined the publicly available datasets R2: Genomics Analysis and Visualization Platform (http://r2.amc.nl). For head and neck SCC the Kaplan–Meier overall survival curve revealed a significant 32-month reduction in the 0.5 overall survival probabilities of patients with high La mRNA expression, when compared to patients with low La mRNA expression (Figure 2A). Similarly, lung adenocarcinoma data showed a significant 8-month reduction in the 0.5 overall survival probabilities of patients with high La mRNA expression, when compared to patients with low La mRNA expression (Figure 2B). 

Taken together, both the La protein and its mRNA expression are elevated in cancer tissue. The overall survival curves showed that in both cancer entities the patients’ survival probability was significantly reduced when La mRNA levels were elevated. These data further accentuate the necessity to understand the functional role of elevated La expression in cancer pathobiology. 

**Figure 3:**
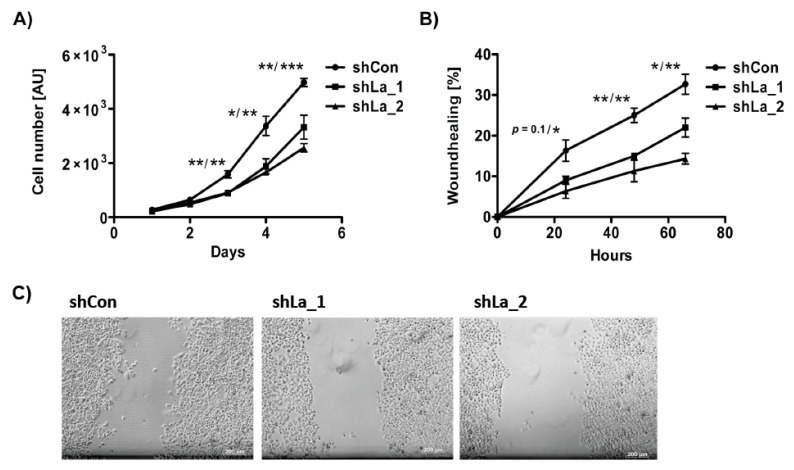
Proliferation and migration is reduced in La-depleted lung adenocarcinoma A549 cells. La depletion was performed by expression of two independent shRNAs (shLa_1, shLa_2) compared to control shRNA (shCon). (**A**) Proliferation and (**B**) wound healing assays, AU = arbitrary units. (**C**) Representative images of control- and La-depleted lung adenocarcinoma A549 cells 24 h after wound scratching. Scale bar 200 µm. (*p* value: <0.05 (one asterisk), <0.005 (two asterisks), <0.001 (three asterisks), *n* = 3).

As shown previously, depletion of the La protein reduces unrestricted cancer cell proliferation of cancer cell lines originating from different cancer entities, including cervical, prostate, and head and neck cancer [8,9]. Concordantly, shRNA-mediated La depletion significantly diminished proliferation of lung cancer cells (Figure 3A). Furthermore, our wound healing assays demonstrated that migration was reduced in La-depleted lung cancer cells (Figure 3B,C), which confirms data obtained in head and neck cancer cells studies completed earlier [8]. Taken together, consistently with findings in head and neck cancer, the La protein is overexpressed in lung cancer tissue, elevated La mRNA expression correlates with poor overall survival of lung cancer patients, and cancer-associated La protein is required for proliferation and migration of lung cancer cells.

### 2.2. TGFβ-Induced Epithelial to Mesenchymal Transition Is Dependent on the La Protein 

**Figure 4:**
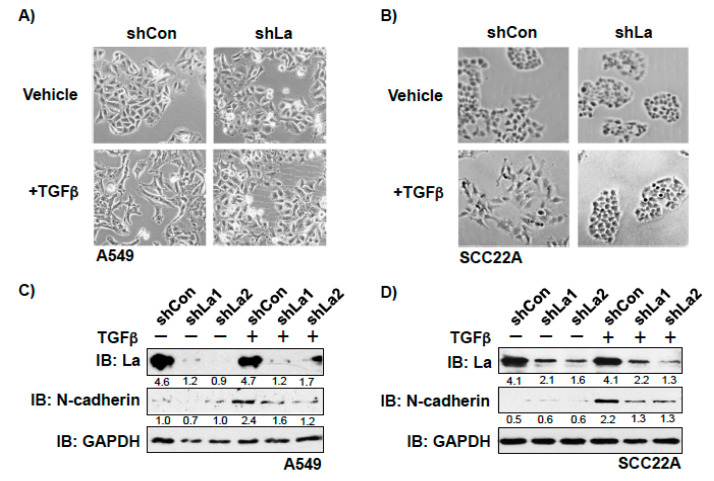
Depletion of La reduces transforming growth factor β (TGFβ)-induced EMT in lung and head and neck cancer cells. La depletion blocks the TGFβ-induced transition from epithelial to mesenchymal phenotype in (**A**) lung adenocarcinoma A549 and (**B**) head and neck squamous cell carcinoma SCC22A cells (representative images of at least three independent experiments (*n* = 3), scale 200-fold magnification). Reduced expression of EMT marker N-cadherin in TGFβ-treated and La-depleted (**C**) A549 and (**D**) SCC22A cells. IB: immunoblot. TGFβ (plus) [5 ng/mL] or vehicle (minus) [1 mg/mL BSA in 4 mM HCl] treatment. Numbers below the bands represent the protein expression normalized to GAPDH.

**Figure 5:**
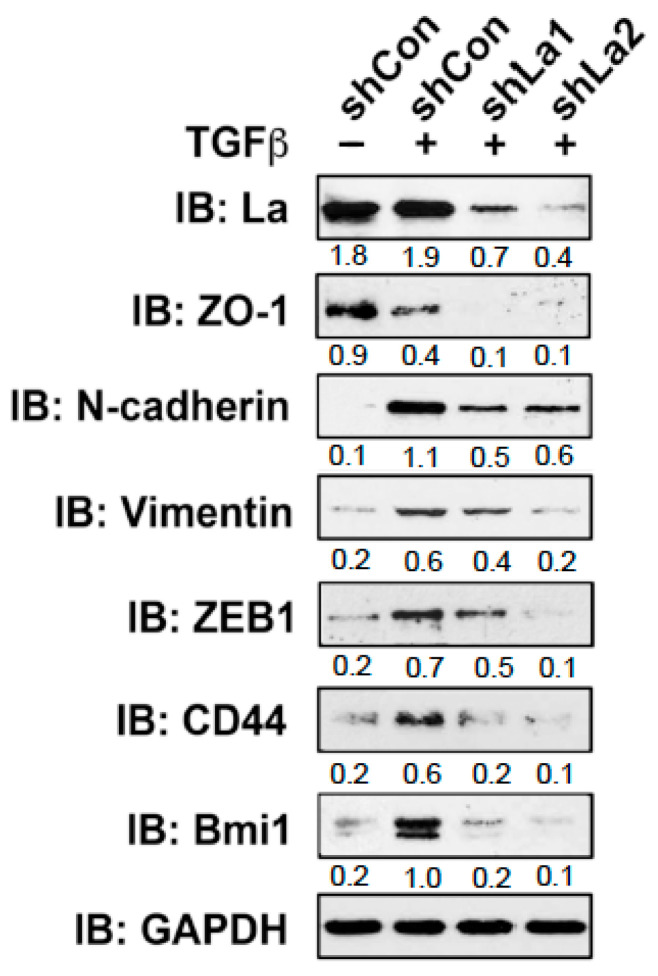
Reduced expression of cellular markers defining EMT and cancer cell stemness in TGFβ-treated and La-depleted cells. Depletion of La reduces the expression of EMT marker ZO-1, N-cadherin, Vimentin, and ZEB1. In addition, the cancer cell stemness marker CD44 and Bmi1 are diminished in TGFβ-treated and La-depleted SCC22A cells. IB: immunoblot. TGFβ (plus) [5 ng/mL] or vehicle (minus) [1 mg/mL BSA in 4 mM HCl] treatment. Numbers below the bands represent the protein expression normalized to GAPDH.

To investigate whether the RBP La plays a role in cancer cell plasticity, we studied TGFβ-induced epithelial to mesenchymal transition (EMT) in control and La-depleted cancer cells. Treatment with TGFβ [5 ng/mL] for 72 h induced transition of a cobblestone-like, epithelial to a fibroblastic or mesenchymal phenotype in control-treated but not in La-depleted cancer cells, as shown for A549 lung cancer cells (Figure 4A and Figure S3) and SCC22A head and neck cancer cells (Figure 4B). Immunoblot analyses confirmed that the TGFβ-induced transition from the epithelial to mesenchymal phenotype was paralleled by increased expression of EMT marker N-cadherin in control-treated but not La-depleted A549 cells (Figure 4C) and SCC22A cells (Figure 4D). Due to the pronounced EMT phenotype in SCC22A cells, we decided to focus on SCC22A cells for further studies, aiming to test our hypothesis that the La protein contributes to cancer cell plasticity. A more in-depth analysis of TGFβ-treated SCC22A cells revealed—as expected for cancer cells undergoing EMT—reduced expression of epithelial marker zonula occludens-1 (ZO-1) paralleled by augmented protein expression of mesenchymal marker N-cadherin, EMT marker vimentin, and transcriptional EMT master regulator ZEB1 in control-treated but not La-depleted cancer cells upon TGFβ treatment (Figure 5 and Figure S3). These data clearly demonstrate that the La protein mediates TGFβ-induced EMT.

### 2.3. The La Protein Supports a Cancer Stem Cell-Like Phenotype

During EMT, cancer cells often gain cancer stem cell (CSC) properties, which are characterized by an increased expression of CSC markers and the ability to grow under serum-free conditions in ultra-low attachment plates as three-dimensional (3D) spheres. First, we tested whether the expression of two CSC markers, namely cluster of differentiation 44 (CD44) and B lymphoma Mo-MLV insertion region 1 homolog (Bmi1) [24], are changed in TGFβ- and control-treated cells. Immunoblot analysis showed that the expression of CD44 and Bmi1 was significantly increased in TGFβ-treated compared to control-treated cells (Figure 5 and Figure S5). In contrast, in TGFβ-treated and La-depleted cells the expression of both CSC markers was distinctly reduced, suggesting that the expression of RBP La supports CSC properties in cancer cells that had undergone TGFβ-induced EMT. 

**Figure 6:**
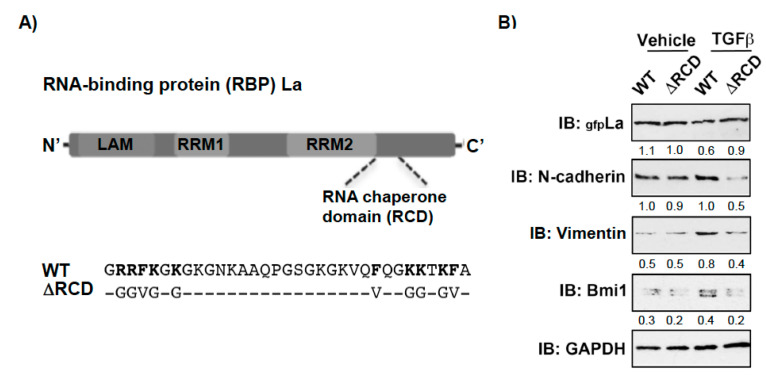
Mutation of the RNA chaperone domain (RCD) activity blocks the rescue of TGFβ-induced, La-dependent EMT and cancer stem cell marker expression in La-depleted cells. (**A**) Scheme of La wildtype (WT) and RNA chaperone domain activity-defective mutant (∆RCD). (**B**) Transient expression of green fluorescence (gfp)-tagged La∆RCD was reduced, but LaWT rescued the expression of mesenchymal marker N-cadherin, EMT marker vimentin, and cancer stem cell marker Bmi1 in TGFβ-treated SCC22A cells. IB: immunoblot. TGFβ (plus) [5 ng/mL] or vehicle (minus) [1 mg/mL BSA in 4 mM HCl] treatment (*n* = 2). Numbers below the bands represent the protein expression normalized to GAPDH.

Recently, we identified a novel RNA chaperone domain (RCD) in the carboxyterminal part of the La protein (Figure 6A and Figure S4) and showed that a RCD activity-defective La mutant (LaΔRCD, Figure 6A) cannot assist the restructuring of RNA stem-loops or increase the expression of reporters regulated by highly structured 5′UTRs [16]. Hence, to test whether the RNA chaperone activity of La is also required for the expression of EMT and CSC markers, we overexpressed gfp-tagged La wildtype (LaWT) and the RCD activity-defective La mutant (LaΔRCD) in La-depleted cells. Strikingly, the TGFβ-induced expression of mesenchymal marker N-cadherin, EMT marker vimentin, and cancer stem cell marker Bmi1 was rescued by transient expression of La wildtype (LaWT) but not by the RCD activity-defective La mutant (LaΔRCD) (Figure 6B). Taken together, these data implicate that the RNA chaperone activity of La contributes to TGFβ-induced EMT and increased expression of CSC markers. 

**Figure 7:**
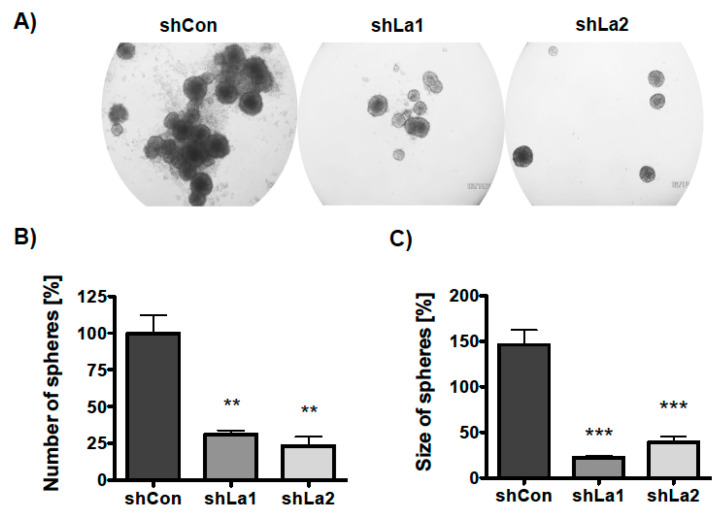
Depletion of La reduces SCC sphere formation and growth. Control- (shCon) or La-depleted (shLa1, shLa2) SCC22A cells were plated in a serum-free medium plus TGFβ [5 ng/mL] on ultra-low attachment plates. (**A**) Representative phase contrast images of spheres, 50-fold magnification. (**B**) Number and (**C**) size of spheres were analyzed after 6 days (*p* value < 0.005 (two asterisks), *p* value < 0.001 (three asterisks), *n* = 3).

Next, we examined whether the La protein supports three-dimensional (3D) sphere formation and growth. Control or La-depleted SCC22A cells were plated in ultra-low attachment plates in the presence of TGFβ or a vehicle-supplemented serum-free medium. The number and size of spheres was determined on day six of culture. Phase contrast microscopic images demonstrated that the La protein is required for sphere formation and growth (Figure 7A). Quantification revealed a significant decrease in the number of spheres formed (sphere formation) by La-depleted SCC22A cells (Figure 7B). Furthermore, the size of spheres (sphere growth) was significantly diminished (Figure 7C), suggesting that the proliferation defect mediated by La depletion contributes to reduced sphere growth. Taken together, the data indicate that the RBP La supports the formation and growth of spheres, a well-known hallmark of a CSC phenotype. However, it remains an open question whether La conveys the positive effect on sphere formation and growth in a direct or indirect manner. 

### 2.4. TGFβ Treatment Induces AKT-Mediated Phosphorylation of La at Threonine 389

The RBP La is post-translationally modified by phosphorylation [7,25] and sumoylation [26,27]. Recently, we described a novel PTM and demonstrated phosphorylation of recombinant La at threonine 389 (T389) by AKT in an in vitro phosphorylation assay [16]. Furthermore, La wildtype but not La harboring a mutation in the phosphorylation site (La^T389A^) enhances cyclin D1 internal ribosome entry site (IRES)-mediated reporter assay translation in cells. To continue to study the role of T389 phosphorylation on endogenous La (pLa^T389^), we applied a pLa^T389^-specific antibody. The specificity of the antibody detecting LaT389 phosphorylation was validated in La-depleted cells and with recombinant La protein by immunoblot analysis (not shown). 

**Figure 8:**
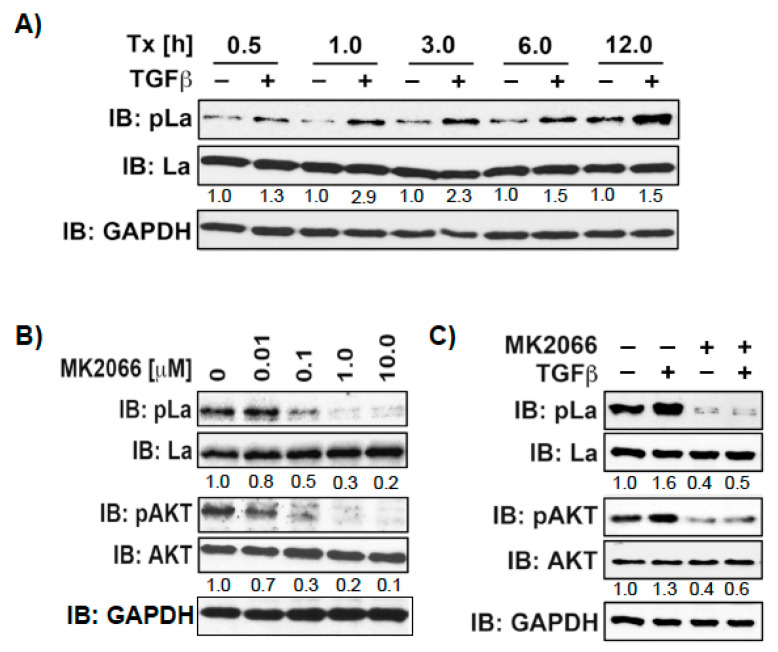
TGFβ treatment increases phosphorylation of La at T389. (**A**) SCC22A cells cultured in serum-free medium were treated with TGFβ [5 ng/mL] or vehicle [1 mg/mL BSA in 4 mM HCl] for increasing time intervals from 0.5 to 12h. (**B**) SCC22A cells cultured in serum-free medium were treated with increasing MK2066 concentrations [0–10 µM] for 3 h followed by insulin treatment [1 µM] for 15 min. (**C**) SCC22A cells cultured in serum-free medium were treated with MK2066 [10 µM] or vehicle for 3 h followed by treatment with TGFβ [5 ng/mL] or vehicle [1 mg/mL BSA in 4 mM HCl] for 1 h. IB: immunoblot. Numbers below bands show the phosphorylation status of La or AKT by calculation the ratio of pLa^T389^/La or pAKT^S473^/AKT. The untreated control was set to 1 in relation to all other samples. GAPDH served as loading control.

Above, we have shown that La promotes TGFβ-induced EMT. Several signaling pathways respond to TGFβ, such as the canonical SMAD pathway, but also noncanonical pathways including AKT, ERK, and MAPK [28]. Therefore, it is reasonable to speculate that TGFβ triggers AKT phosphorylation of La during EMT. To test this assumption, we treated SCC22A cells with TGFβ or vehicle and harvested cell lysates at different time points. Our studies showed that TGFβ increases T389 phosphorylation over time, starting with a slight pLa ^T389^ signal at 0.5 h, which strongly increased over 12 h (Figure 8A and Figure S5). To test whether AKT stimulation correlates with T389 phosphorylation, we treated cells with insulin, a classical activator of AKT, in the presence of increasing pan-AKT inhibitor MK2066 concentrations. As expected, insulin stimulation induced AKT activation, as shown by AKT phosphorylation at serine 473 (pAKT^S473^), which was inhibited by increasing MK2066 concentrations (Figure 8B). Interestingly, increasing MK2066 concentration concurrently reduced pLa^T389^ phosphorylation. Further analysis revealed that TGFβ-induced phosphorylation of La^T389^ correlates with phosphorylation of AKT^S473^ and that TGFβ-induced phosphorylation of both target proteins were inhibited by MK2066 (Figure 8C). Taken together, these data support the notion that TGFβ treatment triggers AKT-mediated phosphorylation of La at T389.

**Figure 9:**
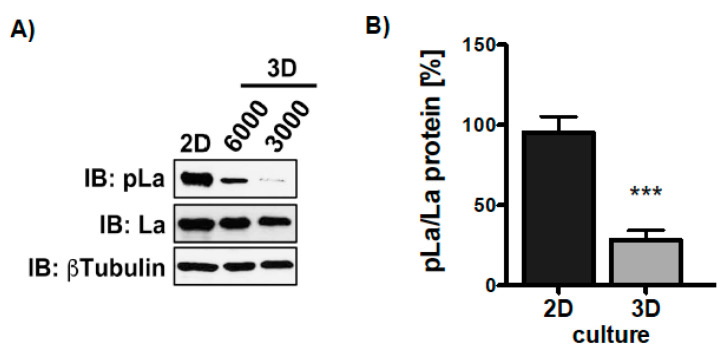
More phosphorylation of La at T389 in 2D compared to 3D cultures. (**A**) Immunoblot (IB) of SCC22A cells 2D-cultured compared to SCC22A cells (3000 or 6000 cells plated) cultured on ultra-low attachment plates (3D sphere culture) in serum-free medium plus TGFβ [5 ng/mL]. (**B**) Quantitative analysis of the pLa^T389^/La protein ratio in 2D versus 3D cultures of SCC22A cells. The βTubulin served as loading control (*p* value < 0.001 (three asterisks), *n* = 3).

Knowing that TGFβ triggers La phosphorylation in 2D cultures, we were eager to find out whether the growth transition from adherent to ultra-low attached sphere growth changes the phosphorylation status of La^T389^. Hence, we compared the phosphorylation status of La in two-dimensional (2D) attachment cultures with 3D sphere cultures. Interestingly, although TGFβ was present in both settings immunoblot analysis revealed that La phosphorylation at T389 was significantly reduced in 3D sphere cultures compared to 2D attachment cultures (Figure 9A,B and Figure S6). In summary, TGFβ triggers AKT-mediated phosphorylation of La^T389^ in 2D but not 3D cultures, suggesting a different functional state of La in adherent cells and spheres. 

## 3. Discussion

Taken together, this study indicates that the RBP La is required for TGFβ-induced EMT and a cancer stem cell-like phenotype of cancer cells. The RNA chaperone domain of the La protein appears to have a pivotal function during this process as suggested by rescuing mesenchymal and CSC protein marker expression (N-cadherin, vimentin, Bmi1) with La wildtype but not RNA chaperone domain activity-defective La mutant protein. Furthermore, TGFβ treatment induces AKT-dependent phosphorylation of RBP La at T389—a novel posttranslational modification present in cancer cells growing in 2D attachment, but not 3D sphere cultures. However, the underlying molecular mechanism remains elusive and current work aims to clarify the role of the RNA chaperone La and its T389 phosphorylation status in cancer cell plasticity.

Although EMT is primarily regulated at the transcriptional level, recent studies suggest that RBP-regulated translation of selective target mRNAs is of importance as well [28,29,30]. For example, binding of RBP hnRNPE1 and translation elongation factor eEF1A1 to the TGFβ-activated translational (BAT) RNA element blocks the translational elongation of target mRNAs and is released under TGFβ-induced EMT [31]. Furthermore, the overexpression of RBP YB1 in noninvasive breast cancer cells induces EMT and activates IRES-dependent translation of SNAIL1, a transcriptional master regulator of EMT [32,33]. Moreover, during EMT of murine hepatocellular carcinoma cells, the RBP La stimulates IRES-dependent translation of laminin B1, a critical extracellular matrix protein [13]. Those studies suggest that during EMT certain RNA-binding proteins can activate the translation of selective target mRNAs and thereby stimulate cancer progression. Along those lines, our study supports the hypothesis that RBP La stimulates the expression of transcriptional master regulator ZEB1 during EMT. La depletion reduced TGFβ-induced EMT and the expression of ZEB1 protein, but ZEB1 mRNA level were unchanged (data not shown), suggesting that the RNA chaperone La controls ZEB1 expression post-transcriptionally. Interestingly, a predicted RNA folding of the ZEB1 5′UTR revealed a stem-loop structure enclosing the authentic translation start site. Similar to studies on La in cyclin D1 and Bcl2 mRNA translation [9,14], we envision that the RNA chaperone activity of La is critical for efficient ZEB1 mRNA translation by unwinding impeding secondary structures and thereby supporting effective translational initiation. 

Recent work suggests that cellular functions of La are regulated by post-translational modifications. For example, we have shown that a small ubiquitin-like modifier (SUMO) of rat La regulates the retrograde transport in axons [34] and facilitates the RNA-binding activity of human La in in vitro and cell-based assays [26,27]. Moreover, phosphorylation of human La at serine 366 (pLa^S366^) by CK2 is the best-studied modification of the human La protein so far. It has been shown that pLa^S366^ localizes within the nucleoplasm, whereas nonphosphorylated La resides in the cytoplasm and nucleolus [7,25]. Studies suggest that phosphorylation of La at serine 366 regulates the binding and translation of 5′-terminal oligopyrimidine (TOP) mRNAs encoding ribosomal proteins and translation elongation factors [35]. In case of murine La, it has been demonstrated that AKT-mediated phosphorylation at threonine 301 induces the shuttling of RBP La from the nucleus to the cytoplasm allowing the protein synthesis of oncogenic target mRNAs, contributing to an oncoproteom in glioblastoma cells [36]. In TGFβ-treated mesenchymal hepatocytes, PDGF and downstream MAPK/ERK-signaling regulates the cytoplasmic accumulation of murine La [10]. More recently, we found that human La is phosphorylated at threonine 389 (T389) by AKT in vitro and stimulates cyclin D1 IRES-mediated reporter expression in cell-based assays [16]. The collective finding that AKT-mediated phosphorylation of mouse [36] and human La [16], although at different threonine residues, modulates the translation of target mRNAs is very interesting. The strong increase in pLa^T389^ after TGFβ treatment suggests that the RNA chaperone La fosters TGFβ–induced EMT by facilitating translation of a specific set of oncogenic target mRNAs, which are characterized by RNA structural elements located close to the translational start codon in the 5′UTRs. Since our data show that phosphorylation of La^T389^ is significantly reduced in cancer cells growing as spheres compared to 2D attachment cultures, the RNA chaperone activity might be reduced in cancer cells expressing cancer stem cell properties necessary for metastatic spread. In future studies we will focus on the interplay between post-translational modifications regulating the RNA chaperone activity of La during translation of mRNAs encoding factors regulating cancer cell plasticity and metastasis.

## 4. Materials and Methods 

### 4.1. Cell Culture and ShRNA Transduction

Both the lung cancer cell line A549, purchased from ATCC, and head and neck cancer cell line UM-SCC 22A (SCC22A) [8,37] were expanded and stored in liquid nitrogen. Cell lines were cultured in advanced DMEM (Gibco/Thermo Fisher Scientific, Inc., Waltham, MA, USA) containing 2 mM L-glutamine (Life Technology/Thermo Fisher Scientific, Inc., Waltham, MA, USA), Penicillin-Streptomycin (Gibco), plus 10% FBS. For three-dimensional (3D) sphere cultures, cells were plated in ultra-low attachment 24-well plates (Corning Costar, Corning, NY, USA) in serum-free medium containing TGFβ [5 ng/mL] or vehicle (1 mg/mL BSA in 4 mM HCl) and cultured for 6 days before analysis. All cell lines were tested for mycoplasma contamination by applying MycoSensor PCR Assay kit according to manufacturer’s instructions (Agilent Technologies, Inc., Santa Clara, CA, USA). For La-specific shRNA-mediated depletion lentiviral transduction of MISSION shRNA constructs (TRCN0000062193 and TRCN0000062195) were performed according to the manufacture’s instruction (Sigma-Aldrich, St. Louis, MO, USA). All lentiviral experiments were performed under biosafety level S2 at our laboratory at the Medical University of South Carolina (Charleston, SC, USA).

### 4.2. Transfection of Gfp-Tagged La Plasmids

Green fluorescence protein (gfp)-tagged wildtype La (WT) and RNA chaperone domain activity-defective mutant La (ΔRCD) has been described recently [16]. For transfection a nucleofector was applied according to the manufacturer’s instructions (Nucleofector Kit V, program U-029, AMAXA, Inc., Seattle, WA, USA).

### 4.3. Immunohistochemistry

The immunohistochemical staining was performed as described recently by applying the monoclonal human La-specific 3B9 antibody as detailed described in [8,9] on two distinct lung cancer tissue microarrays (TMA: BC04118 and LC1002, US Biomax, Inc., Derwood, MD, USA). The predominantly nuclear staining of the RBP La is depicted as percentages based on the largest population of benign cells present in normal tissue, respectively, tumor cells present in cancer tissue. 

### 4.4. Proliferation and Wound healing Assay

Proliferation rates were determined as described recently by applying the CyQuant Cell Proliferation Assay Kit (Invitrogen/Thermo Fisher Scientific, Inc., Waltham, MA, USA) [9]. The numbers of reattached cells was determined 5 h after seeding (FU = fluorescence units). Wound healing assay was performed as described previously [8].

### 4.5. Immunoblot Analysis

Cells were lysed and protein was analyzed by SDS-polyacrylamide gel electrophoresis (SDS-PAGE). The following antibodies were applied: monoclonal human La-specific 3B9 antibody [38], anti-N-cadherin (#14215, Cell Signaling Technology, Inc., Danvers, MA, USA), anti-ZO-1 (#8193, Cell Signaling), anti-Vimentin (#5741, Cell Signaling), anti-CD44 (#3570, Cell Signaling), anti-Bmi1 (#5856, Cell Signaling), anti-ZEB1 (#3398, Cell Signaling), anti-AKT (#4591, Cell Signaling), anti-pAKT (#4060, Cell Signaling), anti-GAPDH (#25778, Santa Cruz Biotechnology, Inc., Dallas, TX, USA), and anti-tubulin (#5568, Cell Signaling). The affinity-purified polyclonal rabbit anti-pLa^T389^ against threonine 389 phosphorylated human La peptide (amino acids 381–393) was generated by YenZym antibodies, LCC, Brisbane, CA, USA. Secondary antibodies were horseradish peroxidase-conjugated (Dianova, Inc., Pine Bush, NY, USA).

### 4.6. Statistical Considerations

Two-tailed *p*-value was determined by *t*-test, applying Prism (Version 5, GraphPad Software, San Diego, CA, USA). *p*-value < 0.05 (one asterisks), <0.005 (two asterisks), and <0.001 (three asterisks). Error bars represent the mean +/− SD of triplicates in n-independent experiments.

## 5. Conclusions

This study indicates that cancer-associated elevated expression of RBP La is critical for TGFβ-induced EMT and the acquisition of cancer stem cell properties. The RNA chaperone domain of La appears to own a pivotal function during this process as suggested by rescuing vimentin and Bmi1 expression with La wildtype, but not RNA chaperone domain activity-defective La mutant protein. Furthermore, TGFβ treatment induces AKT-dependent phosphorylation of La at T389—a novel posttranslational modification—present in cancer cells growing in 2D adherent but not 3D sphere cultures. Our study suggests that the TGFβ/AKT/pLa^T389^ signaling pathway regulates cancer cell plasticity. Since the underlying molecular mechanism and its implication for the cancer cell plasticity remains elusive, current work aims to clarify the role of the RNA chaperone La and its T389 phosphorylation status during cancer progression and metastasis. 

During cancer progression and metastatic colonization the tumor microenvironment is of key importance. Within the tumor microenvironment secreted cytokines and chemokines act as autocrine and paracrine signals and mediate cross-talk between cancer cells, cancer-associated fibroblasts, immune cells, smooth muscle cells, and endothelial cells. In particular, TGFβ signaling has emerged as key player in the tumor microenvironment triggering tumor growth, metastasis and therapy resistance [39,40]. It will be interesting to address in future studies the open question to which extent the TGFβ/AKT/pLa^T389^ signaling pathway is involved in shaping the tumor microenvironment to drive cancer progression.

Our data emphasize a functional role of the RBP La during cancer progression. Aiming to identify an interventional La inhibitor for anticancer therapy, we received first promising data applying a La-based fluorescence polarization high throughput screening assay to screen a library of small molecular compounds for molecules able to block La:mRNA interactions [41]. As a first hit, we identified a small compound able to selectively block the binding of La to target mRNA containing a stem-loop structure in the 5′UTR (antiapoptotic factor Bcl2) and did not affect binding of La to mRNAs containing 5′UTRs with terminal oligopyrimidine tracts (TOP mRNAs), which have been shown to interact with the RNA-binding protein La such as ribosomal proteins rpL37 and rpL5. Our future studies are warranted to further investigate if the RBP La is a valid target for therapeutic intervention.

## Data Availability

The data presented in this study are openly available in R2: Genomics Analysis and Visualization Platform (http://r2.amc.nl).

## Supplementary Materials

The following are available online at https://www.mdpi.com/2072-6694/13/2/343/s1, Figure S1: Overexpression of RBP La in lung cancer tissue analyzed by cancer stage, Figure S2: Overexpression of RBP La in lung cancer tissue analyzed by sex, Figure S3: Supplemental Figure 4 and Figure 5, Figure S4: Supplemental Figure 6, Figure S5: Supplemental Figure 8, Figure S6: Supplemental Figure 9.

## References

[1] Xu Y.J., Wu W., Han Q., Wang Y.L., Li C.C., Zhang P.P., Xu H.X. (2019). Post-translational modification control of RNA-binding protein hnRNPK function. Open Biol..

[2] Sommer G., Heise T. (2020). Role of the RNA-binding protein La in cancer pathobiology. RNA Biol..

[3] Qin H., Ni H.W., Liu Y.C., Yuan Y.Q., Xi T., Li X.M., Zheng L.F. (2020). RNA-binding proteins in tumor progression. J. Hematol. Oncol..

[4] Maraia R.J., Mattijssen S., Cruz-Gallardo I., Conte M.R. (2017). The La and related RNA-binding proteins (LARPs): Structures, functions, and evolving perspectives. Wiley Interdiscip. Rev. RNA.

[5] Dock-Bregeon A.C., Lewis K.A., Conte M.R. (2019). The La-related proteins: Structures and interactions of a versatile superfamily of RNA-binding proteins. RNA Biol..

[6] Stavraka C., Blagden S. (2015). The La-Related Proteins, a Family with Connections to Cancer. Biomolecules.

[7] Kenan D.J., Keene J.D. (2004). La gets its wings. Nat. Struct. Mol. Biol..

[8] Sommer G., Rossa C., Chi A.C., Neville B.W., Heise T. (2011). Implication of RNA-binding protein La in proliferation, migration and invasion of lymph node-metastasized hypopharyngeal SCC cells. PLoS ONE.

[9] Sommer G., Dittmann J., Kuehnert J., Reumann K., Schwartz P.E., Will H., Coulter B.L., Smith M.T., Heise T. (2011). The RNA-binding protein La contributes to cell proliferation and CCND1 expression. Oncogene.

[10] Petz M., Them N.C., Huber H., Mikulits W. (2012). PDGF enhances IRES-mediated translation of Laminin B1 by cytoplasmic accumulation of La during epithelial to mesenchymal transition. Nucleic Acids Res..

[11] Blewett N.H., Maraia R.J. (2018). La involvement in tRNA and other RNA processing events including differences among yeast and other eukaryotes. BBA-Gene Regul. Mech..

[12] Trotta R., Vignudelli T., Candini O., Intine R.V., Pecorari L., Guerzoni C., Santilli G., Byrom M.W., Goldoni S., Ford L.P. (2003). BCR/ABL activates mdm2 mRNA translation via the La antigen. Cancer Cell.

[13] Petz M., Them N., Huber H., Beug H., Mikulits W. (2012). La enhances IRES-mediated translation of laminin B1 during malignant epithelial to mesenchymal transition. Nucleic Acids Res..

[14] Heise T., Kota V., Brock A., Morris A.B., Rodriguez R.M., Zierk A.W., Howe P.H., Sommer G. (2016). The La protein counteracts cisplatin-induced cell death by stimulating protein synthesis of anti-apoptotic factor Bcl2. Oncotarget.

[15] Holcik M., Korneluk R.G. (2000). Functional characterization of the X-linked inhibitor of apoptosis (XIAP) internal ribosome entry site element: Role of La autoantigen in XIAP translation. Mol. Cell. Biol..

[16] Kuehnert J., Sommer G., Zierk A.W., Fedarovich A., Brock A., Fedarovich D., Heise T. (2015). Novel RNA chaperone domain of RNA-binding protein La is regulated by AKT phosphorylation. Nucleic Acids Res.

[17] Dongre A., Weinberg R.A. (2019). New insights into the mechanisms of epithelial-mesenchymal transition and implications for cancer. Nat. Rev. Mol. Cell Biol..

[18] Ayob A.Z., Ramasamy T.S. (2018). Cancer stem cells as key drivers of tumour progression. J. Biomed. Sci..

[19] Ishiguro T., Ohata H., Sato A., Yamawaki K., Enomoto T., Okamoto K. (2017). Tumor-derived spheroids: Relevance to cancer stem cells and clinical applications. Cancer Sci..

[20] Hao Y., Baker D., ten Dijke P. (2019). TGF--Mediated Epithelial-Mesenchymal Transition and Cancer Metastasis. Int. J. Mol. Sci..

[21] Caramel J., Ligier M., Puisieux A. (2018). Pleiotropic Roles for ZEB1 in Cancer. Cancer Res..

[22] Krebs A.M., Mitschke J., Losada M.L., Schmalhofer O., Boerries M., Busch H., Boettcher M., Mougiakakos D., Reichardt W., Bronsert P. (2017). The EMT-activator Zeb1 is a key factor for cell plasticity and promotes metastasis in pancreatic cancer. Nat. Cell Biol..

[23] Staudacher A.H., Al-Ejeh F., Fraser C.K., Darby J.M., Roder D.M., Ruszkiewicz A., Manavis J., Brown M.P. (2014). The La antigen is over-expressed in lung cancer and is a selective dead cancer cell target for radioimmunotherapy using the La-specific antibody APOMAB(R). EJNMMI Res..

[24] Jakob M., Sharaf K., Schirmer M., Leu M., Kuffer S., Bertlich M., Ihler F., Haubner F., Canis M., Kitz J. (2020). Role of cancer stem cell markers ALDH1, BCL11B, BMI-1, and CD44 in the prognosis of advanced HNSCC. Strahlenther. Onkol..

[25] Intine R.V., Dundr M., Vassilev A., Schwartz E., Zhao Y., Depamphilis M.L., Maraia R.J. (2004). Nonphosphorylated human La antigen interacts with nucleolin at nucleolar sites involved in rRNA biogenesis. Mol. Cell. Biol..

[26] Kota V., Sommer G., Durette C., Thibault P., van Niekerk E.A., Twiss J.L., Heise T. (2016). SUMO-Modification of the La Protein Facilitates Binding to mRNA In Vitro and in Cells. PLoS ONE.

[27] Kota V., Sommer G., Hazard E.S., Hardiman G., Twiss J.L., Heise T. (2018). SUMO Modification of the RNA-Binding Protein La Regulates Cell Proliferation and STAT3 Protein Stability. Mol. Cell. Biol..

[28] Lamouille S., Xu J., Derynck R. (2014). Molecular mechanisms of epithelial-mesenchymal transition. Nat. Rev. Mol. Cell Biol..

[29] Bisogno L.S., Friedersdorf M.B., Keene J.D. (2018). Ras Post-transcriptionally Enhances a Pre-malignantly Primed EMT to Promote Invasion. iScience.

[30] Howley B.V., Howe P.H. (2019). TGF-beta signaling in cancer: Post-transcriptional regulation of EMT via hnRNP E1. Cytokine.

[31] Hussey G.S., Chaudhury A., Dawson A.E., Lindner D.J., Knudsen C.R., Wilce M.C., Merrick W.C., Howe P.H. (2011). Identification of an mRNP complex regulating tumorigenesis at the translational elongation step. Mol. Cell.

[32] Evdokimova V., Tognon C., Ng T., Ruzanov P., Melnyk N., Fink D., Sorokin A., Ovchinnikov L.P., Davicioni E., Triche T.J. (2009). Translational activation of snail1 and other developmentally regulated transcription factors by YB-1 promotes an epithelial-mesenchymal transition. Cancer Cell.

[33] Evdokimova V., Tognon C.E., Sorensen P.H. (2012). On translational regulation and EMT. Semin. Cancer Biol..

[34] van Niekerk E.A., Willis D.E., Chang J.H., Reumann K., Heise T., Twiss J.L. (2007). Sumoylation in axons triggers retrograde transport of the RNA-binding protein La. Proc. Natl. Acad. Sci. USA.

[35] Schwartz E.I., Intine R.V., Maraia R.J. (2004). CK2 is responsible for phosphorylation of human La protein serine-366 and can modulate rpL37 5’-terminal oligopyrimidine mRNA metabolism. Mol. Cell. Biol..

[36] Brenet F., Socci N.D., Sonenberg N., Holland E.C. (2009). Akt phosphorylation of La regulates specific mRNA translation in glial progenitors. Oncogene.

[37] Carey T.E., Kimmel K.A., Schwartz D.R., Richter D.E., Baker S.R., Krause C.J. (1983). Antibodies to human squamous cell carcinoma. Otolaryngol. Head Neck Surg..

[38] Kremerskothen J., Nettermann M., de Bekke A., Bachmann M., Brosius J. (1998). Identification of human autoantigen La/SS-B as BC1/BC200 RNA-binding protein. DNA Cell Biol..

[39] Porcelli L., Iacobazzi R.M., Di Fonte R., Serrati S., Intini A., Solimando A.G., Brunetti O., Calabrese A., Leonetti F., Azzariti A. (2019). CAFs and TGF-beta Signaling Activation by Mast Cells Contribute to Resistance to Gemcitabine/Nabpaclitaxel in Pancreatic Cancer. Cancers.

[40] Batlle E., Massague J. (2019). Transforming Growth Factor-beta Signaling in Immunity and Cancer. Immunity.

[41] Sommer G., Fedarovich A., Kota V., Rodriguez R., Smith C.D., Heise T. (2017). Applying a high-throughput fluorescence polarization assay for the discovery of chemical probes blocking La:RNA interactions in vitro and in cells. PLoS ONE.

