# Supplementary material for: RNA-Binding Protein La Mediates TGFβ-Induced Epithelial to Mesenchymal Transition and Cancer Stem Cell Properties
Source: Cancers (Basel). 2021 Jan 19;13(2):343. doi: 10.3390/cancers13020343 (PMC7832410; doi:10.3390/cancers13020343)
Supplement: Supplementary file 1 [file cancers-13-00343-s001.pdf]

Supplementary Materials: RNA-Binding Protein La Mediates TGFβ-Induced Epithelial to Mesenchymal Transition and Cancer Stem Cell Properties

Tilman Heise and Gunhild Sommer

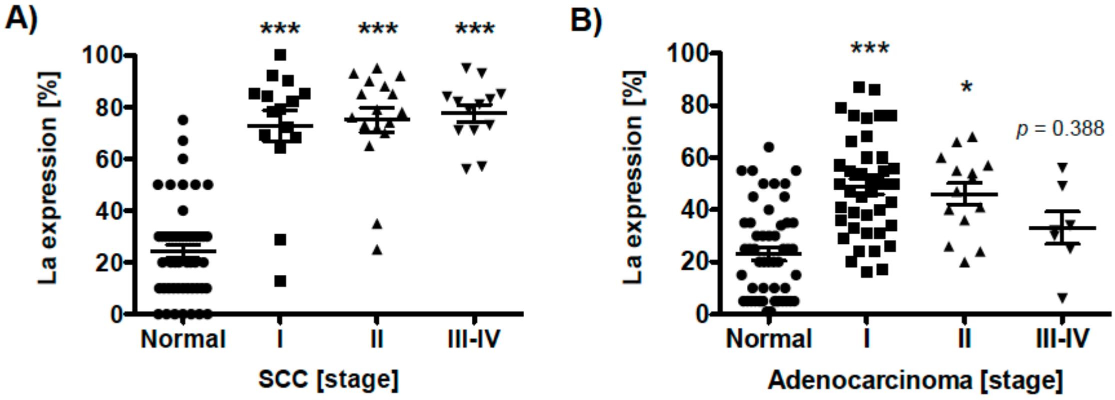

Figure S1. Overexpression of RBP La in lung cancer tissue analyzed by cancer stage. (**A**) SCC (**B**) adenocarcinoma (*p*-value < 0.05 (one asterisk), < 0.001 (three asterisks)).

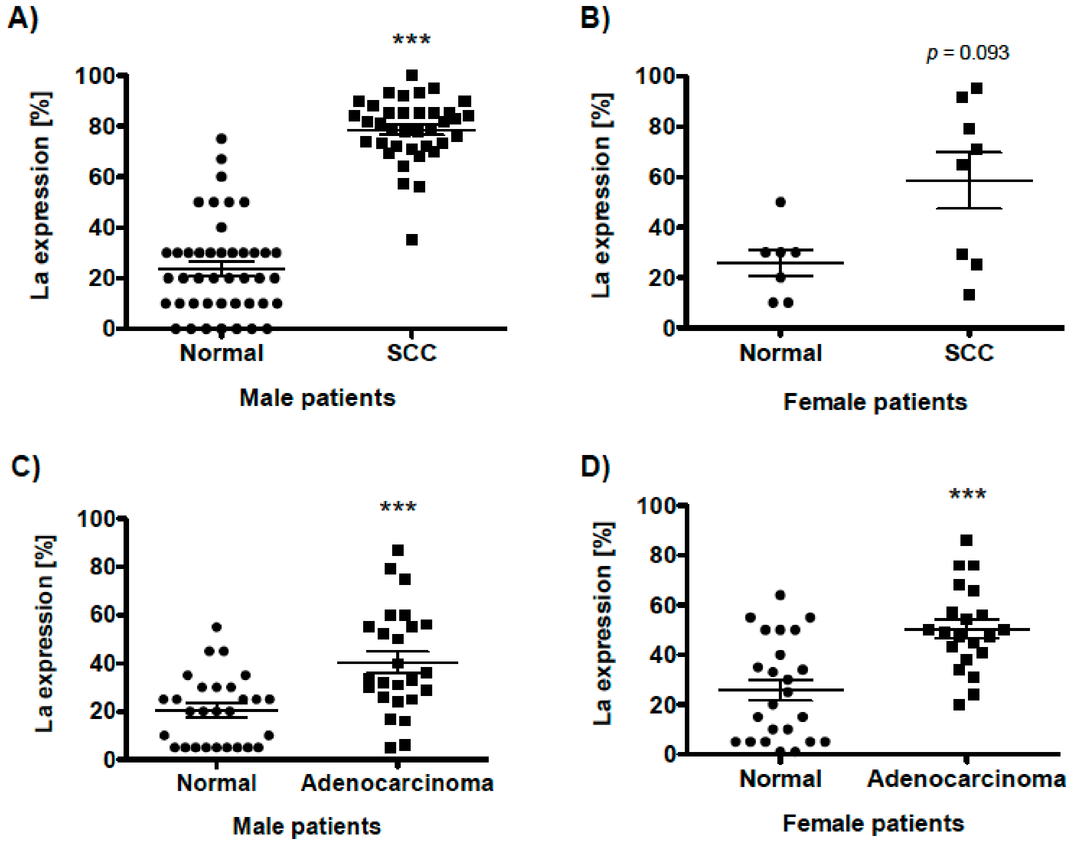

Figure S2. Overexpression of RBP La in lung cancer tissue analyzed regarding sex differences: (**A**) SCC, male, (**B**) SCC, female, (**C**) adenocarcinoma, male, (**D**) adenocarcinoma, female (*p*-value < 0.001 (three asterisks)).

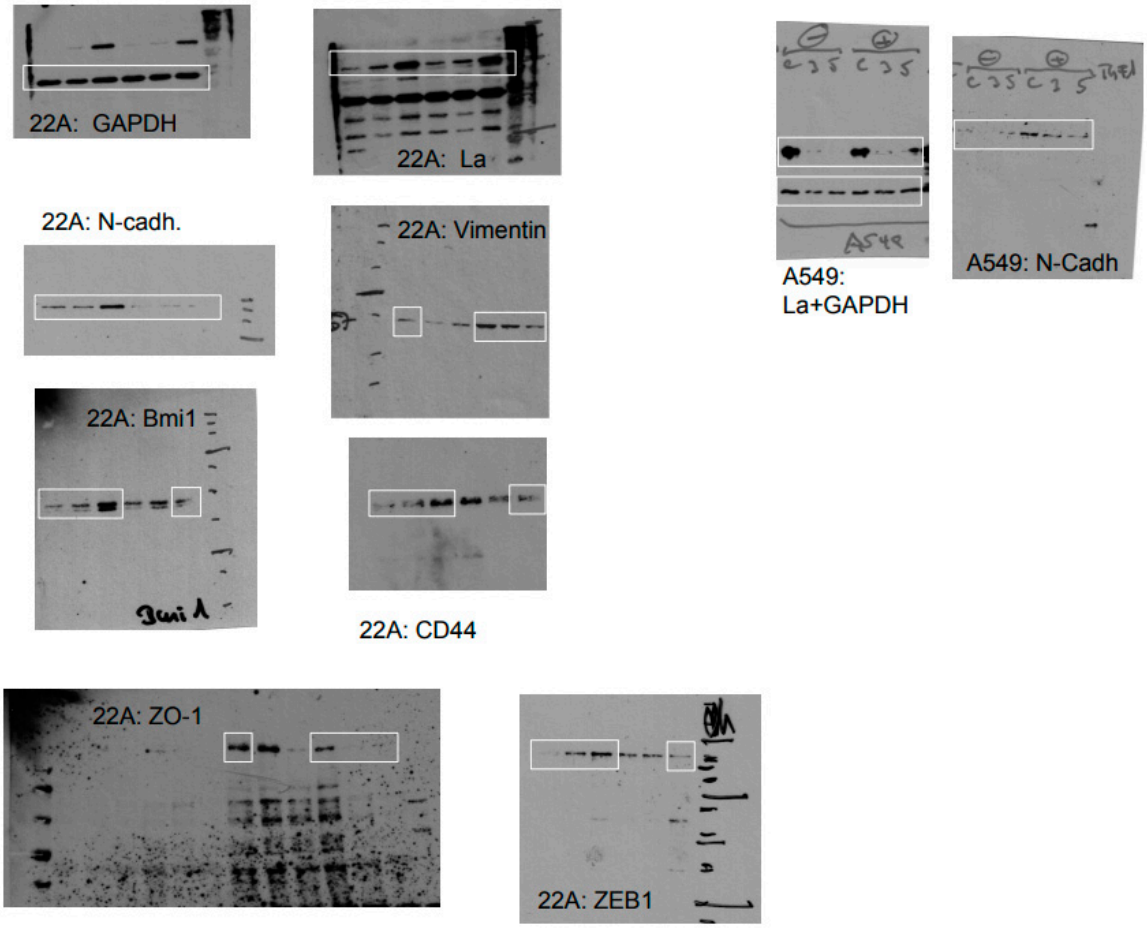

Figure S3. Supplemental Figure 4 and Figure 5.

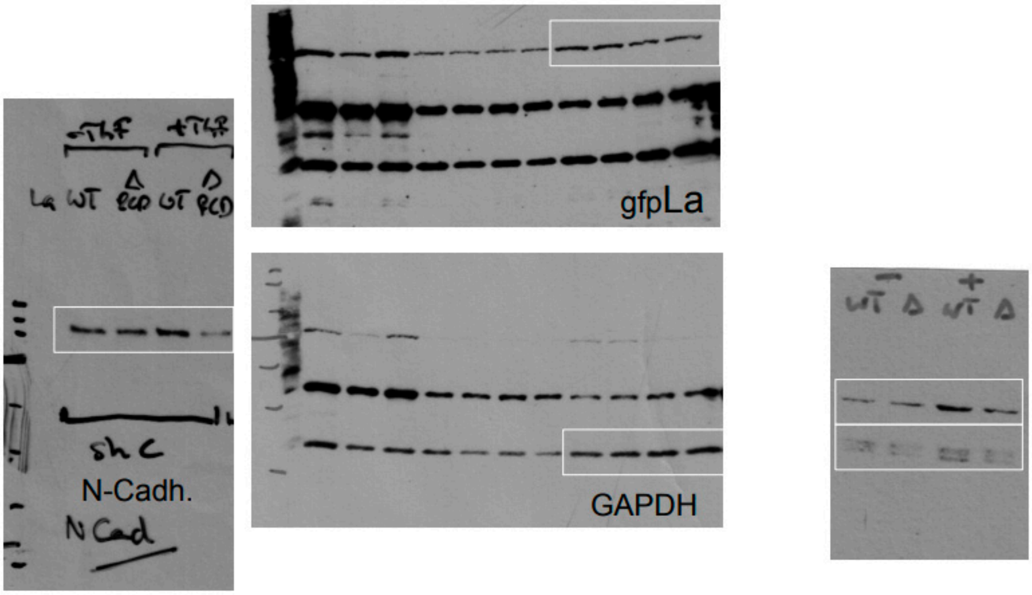

Figure S4. Supplemental Figure 6.

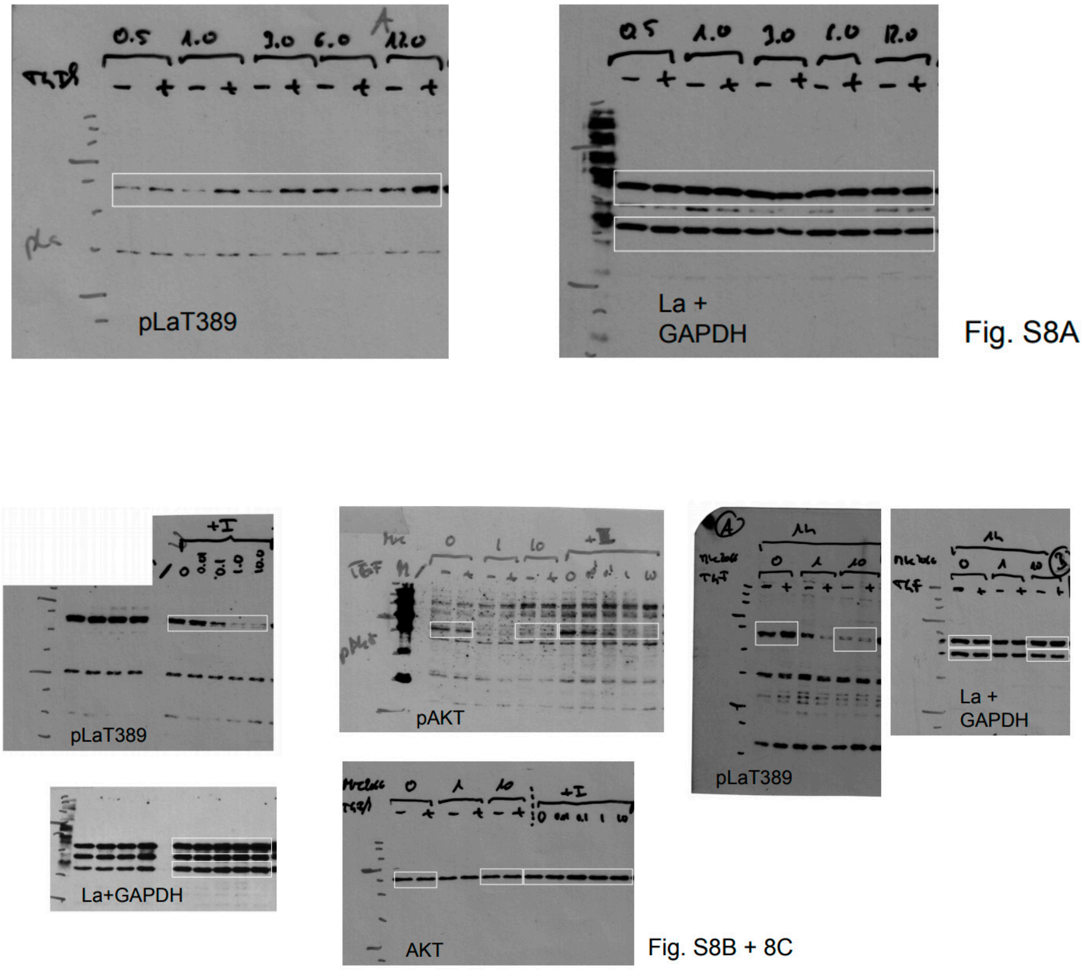

Figure S5. Supplemental Figure 8.

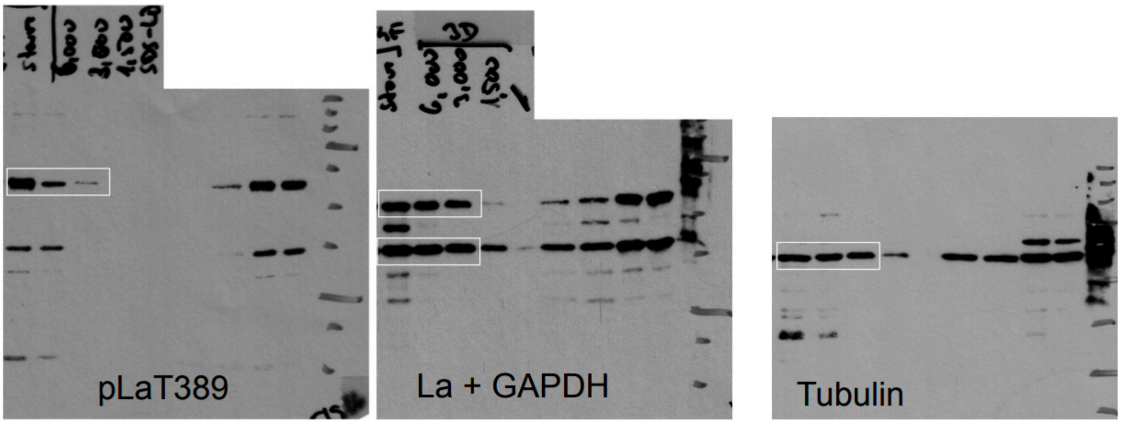

Figure S6. Supplemental Figure 9.
